# Phased nucleotide inserts for sequencing low-diversity RNA samples from in vitro selection experiments

**DOI:** 10.1261/rna.072413.119

**Published:** 2020-08

**Authors:** Devin P. Bendixsen, Jessica M. Roberts, Brent Townshend, Eric J. Hayden

**Affiliations:** 1 Biomolecular Sciences Graduate Programs, Boise State University, Boise, Idaho 83725, USA; 2 Department of Bioengineering, Stanford University, Stanford, California 94305, USA; 3 Department of Biological Sciences, Boise State University, Boise, Idaho 83725, USA

**Keywords:** phased inserts, in vitro selection, sequencing, low-diversity, ribozymes

## Abstract

In vitro selection combined with high-throughput sequencing is a powerful experimental approach with broad application in the engineering and characterization of RNA molecules. Diverse pools of starting sequences used for selection are often flanked by fixed sequences used as primer binding sites. These low diversity regions often lead to data loss from complications with Illumina image processing algorithms. A common method to alleviate this problem is the addition of fragmented bacteriophage PhiX genome, which improves sequence quality but sacrifices a portion of usable sequencing reads. An alternative approach is to insert nucleotides of variable length and composition ("phased inserts") at the beginning of each molecule when adding sequencing adaptors. This approach preserves read depth but reduces the length of each read. Here, we test the ability of phased inserts to replace PhiX in a low-diversity sample generated for a high-throughput sequencing based ribozyme activity screen. We designed a pool of 4096 RNA sequence variants of the self-cleaving twister ribozyme from *Oryza sativa*. For each unique sequence, we determined the fraction of ribozyme cleaved during in vitro transcription via deep sequencing on an Illumina MiSeq. We found that libraries with the phased inserts produced high-quality sequence data without the addition of PhiX. We found good agreement between previously published data on twister ribozyme variants and our data produced with phased inserts even when PhiX was omitted. We conclude that phased inserts can be implemented following in vitro selection experiments to reduce or eliminate the use of PhiX and maximize read depth.

## INTRODUCTION

The development of RNA molecules with desired functions has numerous applications in RNA research. While de novo rational design of sequences that provide a desired function remains an ongoing pursuit (Daher et al. 2017; Weenink et al. 2017), in vitro selection offers a proven experimental approach (Ellington and Szostak 1990; Robertson and Joyce 1990). In vitro selection starts with diverse pools of sequences and uses cycles of functional selection and amplification to enrich only the sequences with the desired function, while discarding unwanted sequences. Nucleotide sequence analysis is often used to monitor the results of in vitro selections. Historically, sequencing has focused on the end-point of selection in order to find a small number of desired sequences that are in high abundance. More recently, advances in high-throughput sequencing have enabled a more quantitative analysis of selections over time and to provide a larger set of functional sequences. High-throughput sequence analysis has been applied to several common goals of in vitro selection, such as finding ligand-specific aptamers (Dupont et al. 2015; Levay et al. 2015), catalytic RNA molecules (Pitt and Ferré-D'Amaré 2010; Ameta et al. 2014; Hayden 2016; Bendixsen et al. 2019; Pressman et al. 2019), and chimeric aptazymes, which are allosteric ribozymes that are engineered by combining aptamer and ribozyme sequences in a single molecule (Martini et al. 2015; Townshend et al. 2015; Kobori et al. 2017). The steps in the process are inspired by evolution, and the cycles of replication, mutation, and selection have also been used to study the process of evolution as it unfolds in real-time in the laboratory (Joyce Gerald 2007; Hayden et al. 2011).

In vitro selections often begin with very diverse pools of sequences (libraries) that may include over 10^14^ different nucleotide sequences. These diverse pools enable the search for rare functions or beneficial combinations of mutations. However, these complex libraries often have no sequence diversity at their 5′ and 3′-ends because these sequence elements are used as primer binding sites for amplification by PCR or reverse-transcription PCR during the regeneration phase of a selection. Importantly, these primer binding sites are the first nucleotides to be sequenced by Illumina platforms, which causes a major challenge when the instrument's automated algorithms are trying to identify the precise location of individual sequence clusters that are subsequently monitored during rounds of sequencing by synthesis (Krueger et al. 2011). Ironically, because of the lack of nucleotide diversity at the beginning of each read, these complex libraries are considered “low-diversity” for the Illumina platforms. Low-diversity samples have been shown to result in poor sequence output and quality. One way to improve the sequencing of samples with low nucleotide diversity is to add randomly fragmented DNA from bacteriophage PhiX (Illumina Technical Report, https://support.illumina.com/bulletins/2017/02/how-much-phix-spike-in-is-recommended-when-sequencing-low-divers.html, April 7, 2017). This PhiX addition changes the balance of juxtaposed fluorescent signals during early sequencing cycles which improves output and quality. However, this PhiX addition also consumes 5%–50% of the sequencing reads, effectively diverting a portion of the sequencing cost toward an unwanted target. For perspective, a typical HiSeq run with 15% PhiX results in the sequencing of the entire PhiX genome approximately 8000 times. In addition to being wasteful, the abundant use of PhiX has also caused contaminated genome assemblies (Mukherjee et al. 2015). A brief survey of the literature indicated that recent in vitro RNA selection experiments used 8%–30% PhiX addition resulting in high-quality data, but with a corresponding sacrifice in read depth, defined as the number of reads assigned to each unique sequence (Kobori et al. 2015, 2017; Kobori and Yokobayashi 2016; Pressman et al. 2017).

The loss of read depth from PhiX can have consequences for the precision and accuracy of functional measurements from sequence data. Several experimental designs use the change in abundance of each unique sequence over selection rounds to quantify function, such as binding affinity or ribozyme activity. The accuracy and precision of this approach depends upon sequencing depth because sequences with lower read depth show much higher variance between replicates (Sims et al. 2014). Therefore, losing a substantial portion of sequencing reads can limit the ability to accurately quantify functions or properties of individual sequences, or fail to detect rare sequences altogether. Alternatively, it is possible to increase the amount of sequences generated by moving to a higher throughput sequencing platform or doing multiple sequencing runs. The cost of more sequencing runs can be a deterrent for many research groups. Another possible approach would be the use of custom read primers during sequencing that bind to primer sequences and shift the starting nucleotides that are sequenced (Caporaso et al. 2012). However, this approach requires coordination with sequencing facilities for testing and optimization.

An alternative approach to improving low-diversity sequencing is to insert nucleotides of variable length and composition when preparing samples for sequencing. This approach has been termed “phasing amplicon sequencing” because it ensures that neighboring clusters are out of phase and no longer produce identical or highly similar fluorescent signals during each sequencing cycle. This is often achieved with a set of PCR primers that each add a different nucleotide sequence upstream of PCR amplicons that will be sequenced. This approach improves sequencing read depth while sacrificing read length, which can be appropriate when molecules are shorter than the read length, and when read depth is important. This approach has been applied to the sequencing of ribosomal genes from microbial communities, and was shown to improve sequence throughput, as well as average base quality scores and effective read length (Hummelen et al. 2010; Wu et al. 2015). This prior work on microbial amplicon sequencing suggests that a similar approach could also improve the sequencing of low diversity cDNA resulting from the in vitro selection of RNA.

**FIGURE 1.**
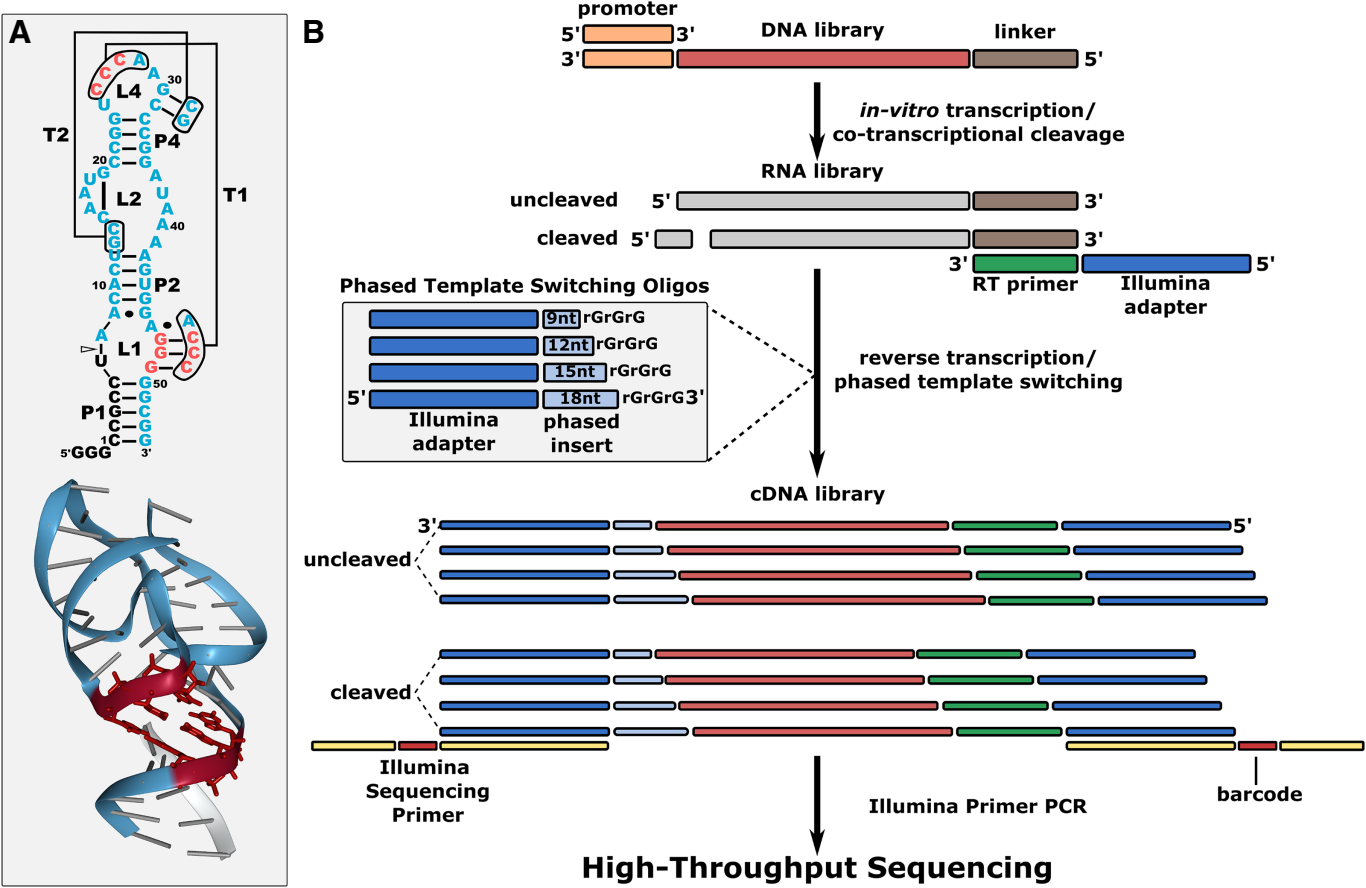
Twister ribozyme library design and in vitro protocol. (*A*) Secondary and tertiary structure of the twister Osa-1-4 ribozyme (Liu et al. 2014; Rose and Hildebrand 2015). The library contained six randomized nucleotide positions indicated by the red nucleotides. The triangle in the secondary structure indicates the cleavage site, and the black nucleotides are the cleaved product. (*B*) Illustration of the protocol for cotranscriptional self-cleavage and phased nucleotide insertion during template switching reverse transcription.The DNA library is ordered as the template strand for transcription, with the T7 promoter at the 3′-end, and a primer binding sequence at the 5′-end (linker). Active variants self-cleave during transcription by T7 RNA polymerase. Cleaved and uncleaved RNA products are reverse-transcribed with template switching using the linker sequence for primer binding, and a pool of four phased template switching oligonucleotides. These phased inserts are incorporated into the the cDNA transcripts during reverse transcription. The resulting single stranded cDNA products with phased inserts are amplified with index primers to add full adaptors for high-throughput sequencing (Illumina).

Here, we apply phasing amplicon sequencing to a high-throughput screening of a library of RNA molecules for their ability to catalyze a self-cleaving ribozyme reaction. We designed an RNA library based on the twister ribozyme from the *Oryza sativa* genome (Roth et al. 2014) with six randomized nucleotide positions in two distal regions that interact in a tertiary structural element (Fig. 1A). This ribozyme cleaves at a sequence-specific position near the 5′-end of the RNA. Assessment of the relative activity of each sequence variant requires sequencing both uncleaved and cleaved molecules in order to quantify the fraction of each sequence that is in the cleaved form (fraction cleaved). To recover the cleaved and uncleaved sequences we used a reverse transcription protocol that relies on the template switching property of the reverse transcriptase to append the sequence of a template switching oligo to the 3′-end of cDNA. This sequence is appended to all RNA molecules, regardless of their 5′-sequence identity, enabling all cDNA to be PCR amplified with the same primer (5′-RACE protocol). To enable phasing amplicon sequencing, we designed four different template switching oligos that each add a different nucleotide sequence immediately after the Illumina sequencing primer binding sight, such that these four unique nucleotide sequences are the first nucleotides sequenced by the Illumina platform. We refer to these unique nucleotide sequences as “phased inserts.” The phased inserts were of four different lengths (9, 12, 15, 18 nt) and were designed to have a balanced nucleotide composition with approximately equal likelihood of A, C, G and T at each of the first nine positions (Fig. 1B), which is expected to improve cluster identification and filtering during these early sequencing cycles. The lengths of these phased inserts were chosen to delay and distribute the sequencing of a five guanosine homopolymer stretch in our library.

To test this approach, we carried out a cotranscriptional cleavage reaction with our ribozyme library and prepared this RNA for Illumina sequencing using the four template switching oligos during reverse transcription, followed by a low cycle PCR to add the full Illumina adaptor. In addition, we prepared a low-diversity control sample without phased inserts, using a single template switching oligo. We then sequenced these two samples on a MiSeq platform under two conditions. The first condition used the addition of 25% PhiX and the second condition used minimal (∼0.5%) PhiX. This minimal amount was added in order to determine sequencing error rates and did not significantly alter nucleotide diversity. We compared these sequencing runs to assess the effectiveness of this phasing amplicon sequencing approach for low-diversity RNA samples.

## RESULTS

### Simulated sequence data predicts that phased inserts improve nucleotide balance

**FIGURE 2.**
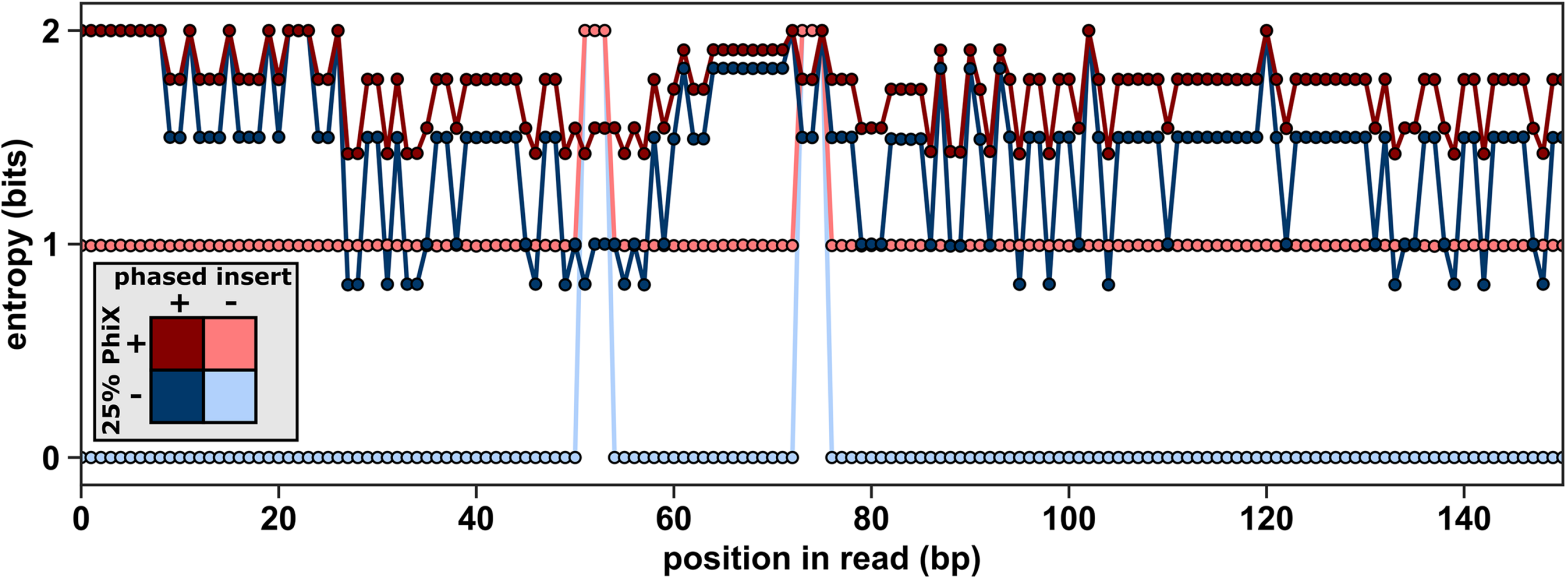
Calculation of positional entropy from simulated sequencing data. Entropy was calculated for four simulated twister ribozyme library samples based on the balance of nucleotides that would be observed at each sequencing cycle. At each position in the read (*y*-axis), the maximum *entropy* = 2 occurs when there is an equal probability of all four nucleotides. The minimum *entropy* = 0 when the same nucleotide occurs at the same position in all sequences. Positional entropy is shown for simulated sequencing runs representing the four conditions that were experimentally sequenced. The control library without phased nucleotide inserts or PhiX (light blue), *only* 25% PhiX (light red), addition of only phased inserts (dark blue), or both phased inserts and PhiX (dark red).

To predict the capacity of our phased inserts to increase the nucleotide diversity of the twister ribozyme samples, we produced simulated sequence data to mimic the sequence diversity expected from the different library preparation protocols. The simulated data used randomly generated collections of one million sequences taken from the twister ribozyme library sequences, then appended our phased inserts, or added random sequences to approximate the PhiX genome, or both. We also simulated a low-diversity control that used only the 4096 unique sequences of the twister library with no phased inserts and no PhiX. These sequences are identical except at the six randomized positions of the T1 pseudoknot. For each of these simulated data sets, we calculated entropy at each position in the sequences from the relative proportion of each nucleotide observed (Fig. 2; Supplemental Fig. S1). Entropy was calculated in units of bits, where an equal probability of all four nucleotides (maximum diversity) at a position has an entropy of two bits, while a single nucleotide (minimum diversity) has an entropy of 0 (Adami 2012). The average positional entropy along the entire 150 nt read was also calculated as a simple comparison metric. The low-diversity control sample produced an average positional entropy of 0.08 ± 0.39 (µ ± σ) (Fig. 2, light blue). The addition of 25% PhiX alone into the sequencing pool produces an increase in the average positional entropy to 1.03 ± 0.19 (Fig. 2, light red). The addition of phased inserts alone (no PhiX) reduces the average positional entropy to 1.43 ± 0.36 (Fig. 2, dark blue), and adding 25% PhiX on top of the phased inserts increases the average positional entropy to 1.73 ± 0.17 (Fig. 2, dark red). Importantly, both phased inserts and PhiX increase the positional entropy over the first 7 nt positions, which are used to map the positions of sequencing clusters in nonpatterned Illumina flow cells currently used in MiSeq. In fact, because the four phased inserts were designed with balanced nucleotides, they increase the entropy to a maximum of two over the first nine positions. We conclude from this simulated sequence data that the phased inserts are expected to increase nucleotide balance beyond PhiX alone suggesting that the addition of PhiX might no longer be needed. The increased diversity resulting from combining both phased inserts and 25% PhiX may provide a solution for the most challenging samples.

### Phased inserts produce high quality sequencing data without PhiX

**FIGURE 3.**
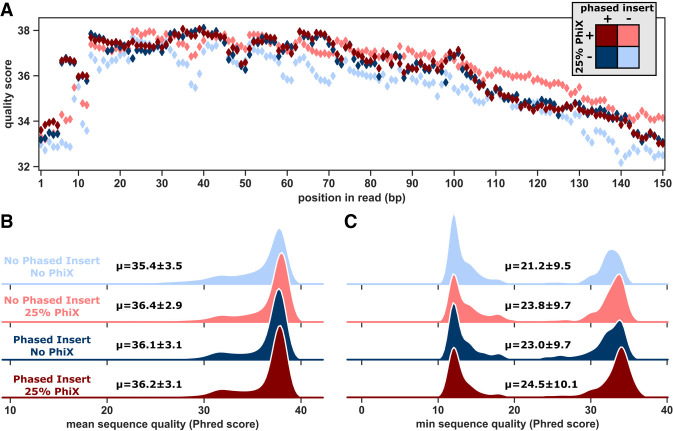
Quality scores of sequencing runs. (*A*) Mean sequencing quality scores per position in the read for samples without phased nucleotide inserts or PhiX (light blue), *only* 25% PhiX (light red), addition of only phased nucleotide insertions (dark blue), or both phased insertions and PhiX (dark red). (*B*) Distribution of mean sequence quality (Phred score) for sequencing runs. (*C*) Distribution of minimum sequence quality (Phred score) for sequencing runs.

To experimentally assess the effectiveness of the phased inserts for this low-diversity ribozyme sample, we compared the data produced by sequencing our phased and un-phased samples with and without PhiX. We determined the percent of clusters passing filter as a measurement of the effectiveness of the phased inserts, because low-diversity samples are expected to have clusters eliminated during early cycles. We also analyzed the quality scores of each sequencing run to ensure that there were no general issues with the samples. We found that our sample with phased inserts had a very high percent of clusters passing filter, even when PhiX was omitted (Table 1). Specifically, with both PhiX and phased inserts, 94% of clusters passed filter. With phased inserts but without PhiX, 91% of clusters passed filter. For the sample without phased inserts, 86% of clusters passed filter when PhiX was included, while only 69% passed filter when PhiX was omitted. All sequencing runs showed similar high-quality data for sequences that passed filter with >90% of base calls greater than Q30 and a mean quality score greater than Q35 (Fig. 3; Supplemental Fig. S2). The results are consistent with our information theory predictions. We conclude that the phased inserts alleviate the loss of clusters when PhiX is omitted, such that a high percentage of clusters passing filter and high quality scores can be achieved with phased inserts alone. While the results indicate that the addition of PhiX can produce slightly higher percentage of clusters passing filter compared to phased inserts alone, it is important to note that ∼25% of these clusters are PhiX genome sequences and do not provide information on ribozyme activity.

**TABLE 1.**
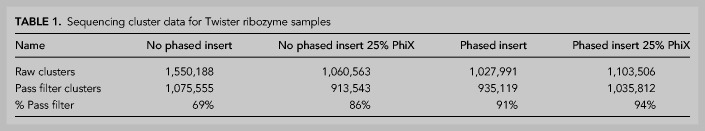
Sequencing cluster data for Twister ribozyme samples

### Comparison of ribozyme activities to previously published data

**FIGURE 4.**
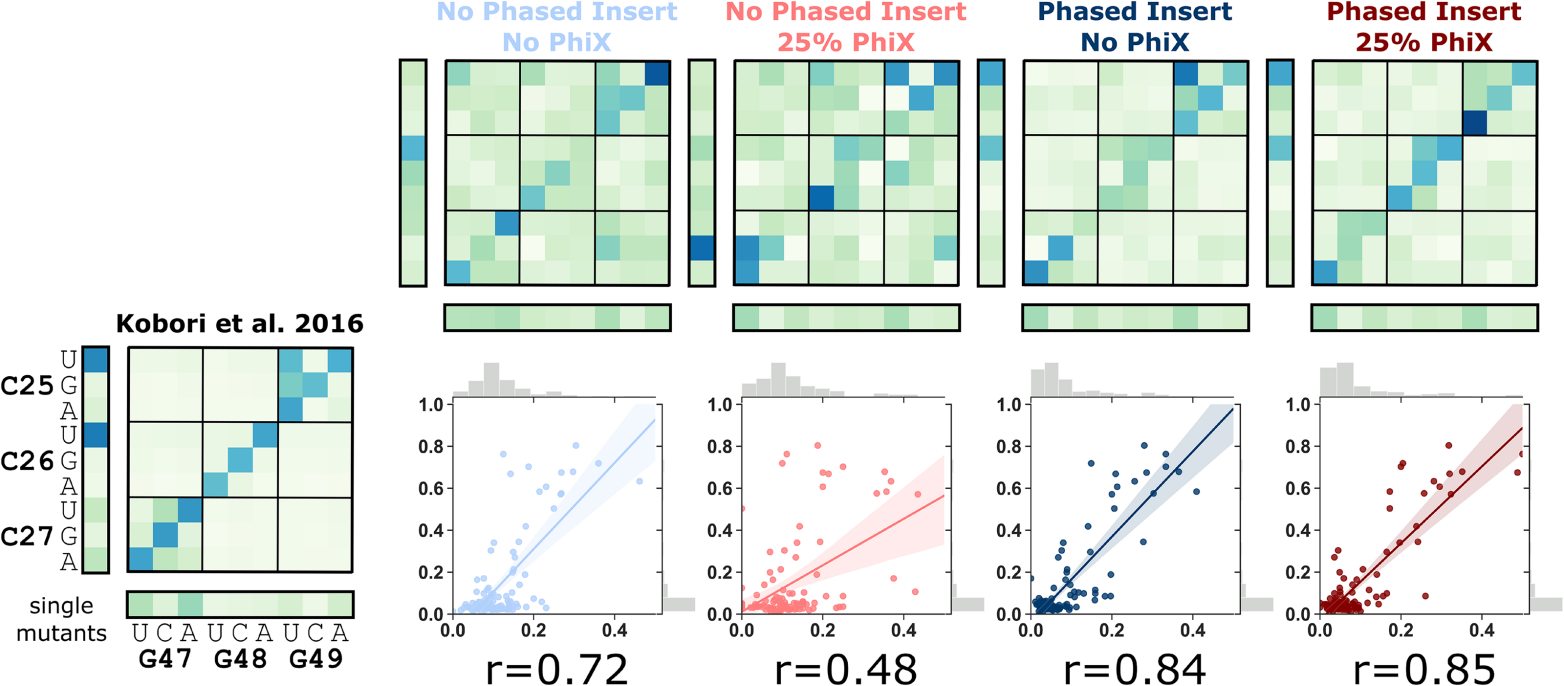
Comparison of ribozyme activities with published data. Heatmap visualization of the activity (fraction cleaved) of the twister ribozyme mutants with previously published values (Kobori and Yokobayashi 2016). Nucleotide identities of mutations are shown as row and column labels. Double mutants are depicted at the intersection of two mutations. The diagonal contains compensatory double mutations that maintain Watson–Crick base pairs. Corresponding heat maps are also shown for the four sequencing runs presented in this study. Correlations between our data and the published values are shown *below* each heatmap. Pearson correlation coefficients (r) are shown *below* each correlation.

Next, we set out to determine whether or not eliminating PhiX had a noticeable effect on the measurement of ribozyme activity in our assay. The relative ribozyme activity of a subset of our ribozyme library was previously reported in a high-throughput sequencing based mutation analysis (Kobori and Yokobayashi 2016). Specifically, the activity for 18 single and 81 double mutants within the T1 pseudoknot were previously reported. The data sets were compared as heatmaps and also by determining the correlation between the activity measured for each ribozyme sequence (Fig. 4). In the heat maps the activities of sequence with a single mutation are on the leftmost column and bottom row. Along the diagonal are the activities of ribozymes with compensatory double mutations that convert one of the C–G base pairs to a different Watson–Crick base pair (G–C, A–U, or U–A). The pseudoknot in the wild-type ribozyme is comprised of three consecutive C–G base pairs. We found a good general agreement between our data and previously reported data. The samples with only phased inserts showed a high correlation (Pearson *r* = 0.84), with only a minor increase by the addition of PhiX (Pearson *r* = 0.85). Both data sets produced without phased inserts showed lower correlation with the previously published data, which we attribute to the lower read depth achieved in this sample (Supplemental Fig. S3). Our assay conditions were slightly different than the previous publication in terms of magnesium concentration, pH and time, which may account for most of the differences in measured activities relative to the previous data. We conclude that omitting PhiX from our phased samples does not dramatically alter the activities measured.

## DISCUSSION

The results demonstrate that the four phased inserts were effective in overcoming the low diversity of our ribozyme-derived amplicon libraries. However, different amplicon libraries may have sequence elements that require unique phasing strategies. The location and length of homopolymer stretches are a known challenge that can lead to low diversity despite phasing. For example, the v4 region of 16S rRNA has multiple stretches of three to five consecutive guanosine residues. Previous phasing amplicon experiments showed that longer stretches of phased nucleotides (0–7 bp) were able to improve the base diversity and sequence quality of the v4 region, while shorter phased inserts were insufficient (Wu et al. 2015). In addition, having unique inserts in multiples of four allows them to be designed with a balance of all four nucleobases within the phased region. Similar to this previous work, we found that our four longer phased inserts (9, 12, 15, and 18 bp) were able to increase base diversity despite a GGGGG homopolymer stretch at the beginning of all uncleaved sequences, which are the majority of reads in our data. Our theoretical and experimental results indicate that this approach was effective for our ribozyme samples. Current cluster evaluation algorithms use the first 25 cycles to filter out low quality clusters (Illumina Document #10000000 71511 v00, March 2019). For some libraries, it may be necessary to increase the phased inserts up to 25 bp to ensure that any long homopolymer stretches occur after sequencing cycle 25. Conversely, it may be possible to reduce the length of phased inserts for libraries lacking homopolymer stretches. If read length is critical, the previously published 0–7 bp phased inserts could be an appropriate solution. Researchers must consider the unique make-up of their library, and the relative importance of read length and read depth in their data when designing the length and composition of phased inserts.

In addition to specific library considerations, researchers using amplicon sequencing must consider how different sequencing technologies will affect their results. Differences in the number of fluorescent dyes (four-color vs. two-color), nonpatterned and patterned flow-cells, instrument optics and raw data analysis algorithms create a large potential phase space. It is unlikely that a single phasing approach will be optimal in all situations. We anticipate that our approach will be most useful to researchers sequencing samples similar to those reported here. Open science practices and data sharing will enable more rigorous meta-analysis of the effectiveness of phasing approaches in the future.

A comparison between our data and previous data on twister ribozyme variants offers some biochemical insights beyond acting as a metric to compare sequencing runs. The previously published data was collected with a longer cotranscription time (2 h), higher pH (8.0) and lower magnesium concentration (6 mM), which all have effects on ribozyme activity. The previous data and our current data are very similar in that pairs of mutations leading to compensatory base pairs tend to maintain high relative ribozyme activity, observed along the diagonal of the heatmaps. The majority of the differences between the previous data and our current data lie off the diagonal. These positions in the heat map represent pairs of mutations that do not result in a Watson–Crick base pair at one position in the pseudoknot. In general, we found higher activity for both G–U and A–C wobble base pairs, suggesting that these base pairs are slightly stabilized by our specific experimental conditions, with higher magnesium concentration being a likely cause of this stabilizing effect. In the future, more systematic comparisons between experiments with different conditions may be used to better understand genotype by environmental interactions for RNA molecules in general.

In conclusion, we find that phased inserts are an effective method to increase the nucleotide diversity of in vitro transcribed RNA samples. The phased inserts can reduce the requirement for PhiX addition, and in some cases may allow elimination, or a minimal addition of ∼1% used for quality assurance. While our study system analyzed self-cleaving ribozymes, our results should hold for other in vitro selection experiments, including selections for RNA and DNA aptamers and aptazymes. We introduced phased inserts during a template switching 5′-RACE protocol, but phased inserts could be introduced using other common methods for RNA sample preparation, such as RNA ligation or subsequent PCR steps. The approach sacrifices some read length for improving read depth, which is appropriate for experiments that require read lengths shorter than the available sequence kit read lengths. Continued research will be required to ensure the effectiveness of amplicon phasing approaches as sequencing technology evolves.

## MATERIALS AND METHODS

### Simulated sequencing run entropy calculation

Information theory was used to calculate the expected entropy at each position based on the alignment of 1 million simulated sequencing reads. At each nucleotide position entropy (*H*) was defined as:

$$H(x)=-\overset{N}{\underset{i=1}{\sum }}p_{i}log_{2}p_{i}$$

(Adami 2012), where *N* = 4 represents the four canonical DNA nucleotides and *p*_*i*_ indicates the relative proportion of that nucleotide at that position. This was repeated for four computationally simulated sequencing libraries: (i) no phased insert + no PhiX, (ii) no phased insert + 25% PhiX, (iii) phased insert + no PhiX, and (iv) phased insert + 25% PhiX.

### Phased insert design

Four template switching oligonucleotides (TSO) were designed with phased inserts that added 9, 12, 15, or 18 nt (Table 2). The phased inserts were designed to have a balance of all four bases between them. The phased nucleotides were inserted between the partial Illumina adapter and three ribose guanines in a TSO needed for template switching reverse transcription. The four phased TSO were quantified by UV absorbance and combined in equal proportions in Tris-EDTA pH 8 at a final concentration of 10 µM total oligonucleotides.

**TABLE 2.**
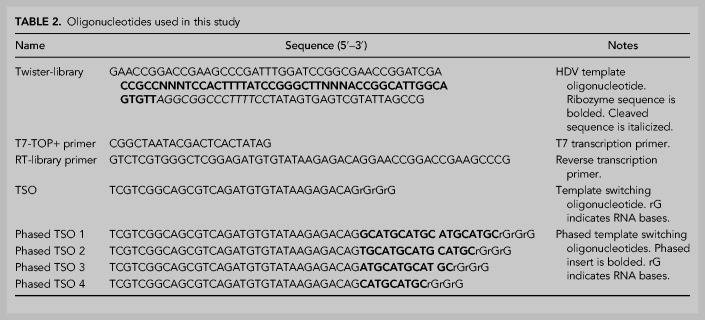
Oligonucleotides used in this study

### Twister library design

The twister library was synthesized as a “machine-mixed” ssDNA oligonucleotide (IDT). The library was synthesized as the reverse complement to act as the template strand during in vitro transcription with T7 RNA polymerase. The minus strand of the T7 promoter was included at the 3′end of the oligo. A fixed sequence (linker) was included at the 5′end of the oligo to serve as a primer binding site for reverse transcription (Table 2; Wilkinson et al. 2006). The ribozyme sequence included 54 nt taken from *Oryza sativa* (Osa-1-4) ribozyme except with randomized bases (N) at six nucleotide positions that correspond to the T1 pseudoknot (Fig. 1A). This results in a library of 4^6^ = 4096 unique RNA sequences.

### Cotranscriptional self-cleavage assay

The promoter region of the ssDNA library was made double stranded by annealing the T7-TOP+ oligo (Table 2). Reactions contained 20 pmol of each oligonucleotide and 10× T7 buffer (300 mM Tris pH 7.5, 500 mM DTT, 200 mM Spermidine, 100 mM MgCl_2_). Oligos were heated to 98°C for 5 min then cooled to room temperature and diluted 10-fold in 1× T7 buffer. Transcription reactions used 8 µL of annealed library in a 200 µL reaction with 1× T7 buffer, 4 µL rNTP (25 mM, NEB), 8 µL T7 RNA polymerase (200 units, Thermo Scientific) and 160 µL RNase free water (Ambion) and were incubated at 37°C for 20 min. The transcription and ribozyme self-cleavage was terminated by the addition of 15 µL of 50 mM EDTA. Protein and buffer were removed using Direct-zol RNA MicroPrep w/TRI-Reagent (Zymo Research). The sample was eluted in 7 µL nuclease free water, quantified by UV absorbance, normalized to 5 µM, and checked for quality by denaturing PAGE (10% polyacrylamide, 8 M urea).

### Reverse transcription with phased template switching

The purified RNA (5 pmol) was mixed with 20 pmol of RT-library primer (Table 2) in a final volume of 10 µL and heated at 72°C for 3 min, then cooled on ice. A 10 µL mixture, consisting of 4 µL SMARTScribe 5× First-Strand Buffer (Clontech), 2 µL dNTP (10 mM), 2 µL DTT (20 mM), 1 µL water and 1 µL SMARTScribe Reverse Transcriptase (100 units, Clontech), were added to the RNA template and RT primer. SMARTScribe Reverse Transcriptase was chosen for its template-switching activity which allows for the addition of a constant primer binding site onto the 3′-end of the cDNA. The phased TSO mix (or nonphased TSO for low diversity control) (20 pmol) was added resulting in a 22 µL reverse transcription reaction. The mixture was then incubated at 42°C for 90 min, followed by heating the mixture to 72°C to stop reverse transcription and degrade the RNA. The single stranded cDNA product was purified using a silica-based column kit (Zymo Research) eluted with 7 µL nuclease free water.

### Illumina adapter PCR and high-throughput sequencing

Illumina adapter sequences were added to each end of the cDNA libraries using low-cycle PCR. The PCR reaction consisted of a 1 µL cDNA library, 12.5 µL KAPA HiFi HotStart ReadyMix (2×, KAPA Biosystems), 2.5 µL forward, 2.5 µL reverse primer (Illumina Nextera Index Kit) and 5 µL water. Each PCR cycle consisted of 98°C for 10 sec, 63°C for 30 sec and 72°C for 30 sec. Multiple PCR cycles were analyzed using gel electrophoresis. A cycle with observable dsDNA but prior to saturation was chosen for sequencing. The PCR products were purified with a silica-based column kit (Zymo Research) and verified using gel electrophoresis. The samples were sent to the University of Oregon Genomics and Cell Characterization Core Facility for quality control, quantification by qPCR and sequencing on the Illumina MiSeq (Nano mode, single end 150 bp). The same samples were run on the MiSeq sequencer twice, once with a 25% PhiX addition and once with a minimal 0.5% PhiX. The 0.5% PhiX does not significantly alter diversity but was added at the request of the core facility in order to ensure similar sequencing error rates between sequencing runs, which is determined by comparing the PhiX reads to the PhiX reference genome.

### Data analysis

Sequencing data were analyzed using Biopython (Cock et al. 2009, 2010) and custom Python scripts (Version 3.7.0). Raw sequencing data are available at the European Nucleotide Archive (ENA accession number: PRJEB33165). Python scripts and data used for analyses are available on GitLab (https://gitlab.com/devinbendixsen/phased_inserts_rna). Relative activity values (*w*) for each unique sequence, or genotype, were determined from the fraction of sequencing reads found in the cleaved form (*N*_*cleaved*_) divided by the total reads of that genotype: *w = N_cleaved_/(N*_cleaved_ + *N_uncleaved_)*. The probability of failed detection of low abundance sequences were determined by sampling from a binomial distribution (Python), with number of trials *n* = 689,334 and the probability of detection *P* = 4.45 × 10^−6^. Probabilities were determined by dividing the number of model iterations that generated zero positive detections by the total number of model attempts (200,000).

## SUPPLEMENTAL MATERIAL

Supplemental material is available for this article.

## Supplementary Material

Supplemental Material
