# Supplementary material for: Phased nucleotide inserts for sequencing low-diversity RNA samples from in vitro selection experiments
Source: RNA. 2020 Aug;26(8):1060–8. doi: 10.1261/rna.072413.119 (PMC7373987; doi:10.1261/rna.072413.119)
Supplement: Supplemental Material [file supp_26_8_1060__index.html]

Phased nucleotide inserts for sequencing low-diversity RNA samples from in vitro selection experiments — Supplemental Material 

# Phased nucleotide inserts for sequencing low-diversity RNA samples from in vitro selection experiments

## Supplemental Material

- Supplemental\_Notes\_Figures.docx
