# Supplementary material for: Phased nucleotide inserts for sequencing low-diversity RNA samples from in vitro selection experiments
Source: RNA. 2020 Aug;26(8):1060–8. doi: 10.1261/rna.072413.119 (PMC7373987; doi:10.1261/rna.072413.119)
Supplement: Supplemental Material [file supp_072413.119_Supplemental_Notes_Figures.docx]

**SUPPLEMENTARY MATERIAL**

**Phased nucleotide inserts for sequencing low-diversity RNA samples from *in vitro* selection experiments**

Devin P. Bendixsen^a,1^, Jessica Roberts^a^, Brent Townshend^b^ & Eric J. Hayden^a,c^

^a^Biomolecular Sciences Graduate Programs, Boise State University, 1910 University Dr., Boise, ID, USA.

^b^Department of Bioengineering, Stanford University, 443 Via Ortega, Stanford, CA, USA

^c^Department of Biological Sciences, Boise State University, 1910 University Dr., Boise, ID, USA.

^1^Present address: Department of Zoology: Population Genetics, Stockholm University, 106 91 Stockholm, Sweden.

**SUPPLEMENTAL NOTES**

*Comprehensive mutational analysis of twister T1 pseudoknot*

In addition to using the twister ribozyme as a model system to validate the benefits of the phased nucleotide inserts, the data also enables a comprehensive evaluation of the T1 pseudoknot. The previously published data included only sequences with one or two mutations in the T1 pseudoknot (154 genotypes). Our current twister library consists of 3,942 additional genotypes, which includes sequences with combinations of 3-6 mutations relative to the wildtype sequence. To understand the relationship between pseudoknot thermodynamic stability and ribozyme activity, we first categorized the genotypes into subpopulations based on the presence of the number of base pairs at the three positions. We plotted the distributions of relative ribozyme activity for each category using our data set obtained without PhiX (Supplemental Fig. S4A). As expected the 64 genotypes that form canonical Watson-Crick base pairs had the highest average relative fitness. This is followed by the three subpopulations that retain two Watson-Crick pairs and a single G-U wobble pair. We note that within this group, the position of the G-U wobble matters. There exists a non-canonical A-A base interaction in T1 that is conserved in >97% of all known twister ribozymes, and which is immediately adjacent to the general base required for the catalytic mechanism (G45) (Wilson et al. 2016). The relative activity of ribozymes decrease on average as the G-U wobble moves closer to the A-A interaction, suggesting that the G-U wobble has a more deleterious effect as it moves closer to the active site. A similar trend was noticed in a randomized stem loop in a HDV-like ribozyme (Kobori and Yokobayashi 2018). As the mismatch mutation came closer to the ribozyme core, the relative activity decreased.

Next, in order to characterize the 64 genotypes that form canonical Watson-Crick base pairs, we calculated the Gibbs free energy for each of these T1 pseudoknots based on nearest-neighbor rules (Turner and Mathews 2010). We plotted ribozyme fitness as a function of free energy (Supplemental Fig. S4B). The plot shows a negative correlation between the measured relative activity and the change in free energy (R^2^ = 0.23, p <0.0001, n =64). This data confirms the importance of the stability of the T1 pseudoknot to the overall ribozyme structure and function (Supplemental Fig. S4B). However, we note that the sequences with the highest ribozyme fitness do not have the lowest free energy. This indicates that specific interactions between each T1 sequence and the rest of the ribozyme are also important.

*PhiX elimination can improve the detection of low abundance reads*

We observed that the ~25% reduction in ribozyme data from PhiX addition had consequences for the detection of low abundance sequences. Specifically, comparing the two sequencing runs of the phased sample we observed 145 more genotypes as cleaved when PhiX was omitted. Rare sequences have a certain probability of not being observed by chance. In fact, using a binomial framework we estimate a high probability that a cleaved genotype will drop out of our data by chance if it was only observed once (p = 0.37) or twice (p = 0.13) in our no PhiX sequence data (899,174 ribozyme reads). However, the probability of a cleaved genotype being missed increases when PhiX is added. With the reduced number of reads mapping to ribozymes in the 25% PhiX data (689,334 ribozyme reads), the probability that genotype observed once or twice will be missed increases to p = 0.47 and p = 0.22, respectively. Genotypes observed as cleaved three to five times in our no PhiX sample are more than twice as likely to be missed when PhiX is omitted. This analysis illustrates how eliminating or reducing PhiX can improve the detection of low abundance reads, which can be important for in vitro selection experiments.

*Failed HiSeq 3000 sequencing run using Hepatitis Delta Virus (HDV) ribozyme*

We previously observed a failed sequencing run when phased inserts were not used. The library was based on the Hepatitis Delta Virus ribozyme which, similar to the twister ribozyme, exhibits 5’ self-cleavage activity. The library was prepared in an identical fashion to the twister library in this study, except without phased inserts in the template switching oligos. The sample was then sequenced using Illumina HiSeq 3000 platform with the addition of 10% PhiX. The run resulted in 12.26% of clusters passing the filter. The sequencing reads that did pass the filter were of low quality and had a mean quality score of 27.9±0.003. Although this library was sequenced on a HiSeq as compared to the MiSeq platform in this study, both platforms are known to have significant issues with low-diversity samples. Furthermore, it is recommended that PhiX be used for both platforms to increase the nucleotide diversity.

**SUPPLEMENTAL FIGURES
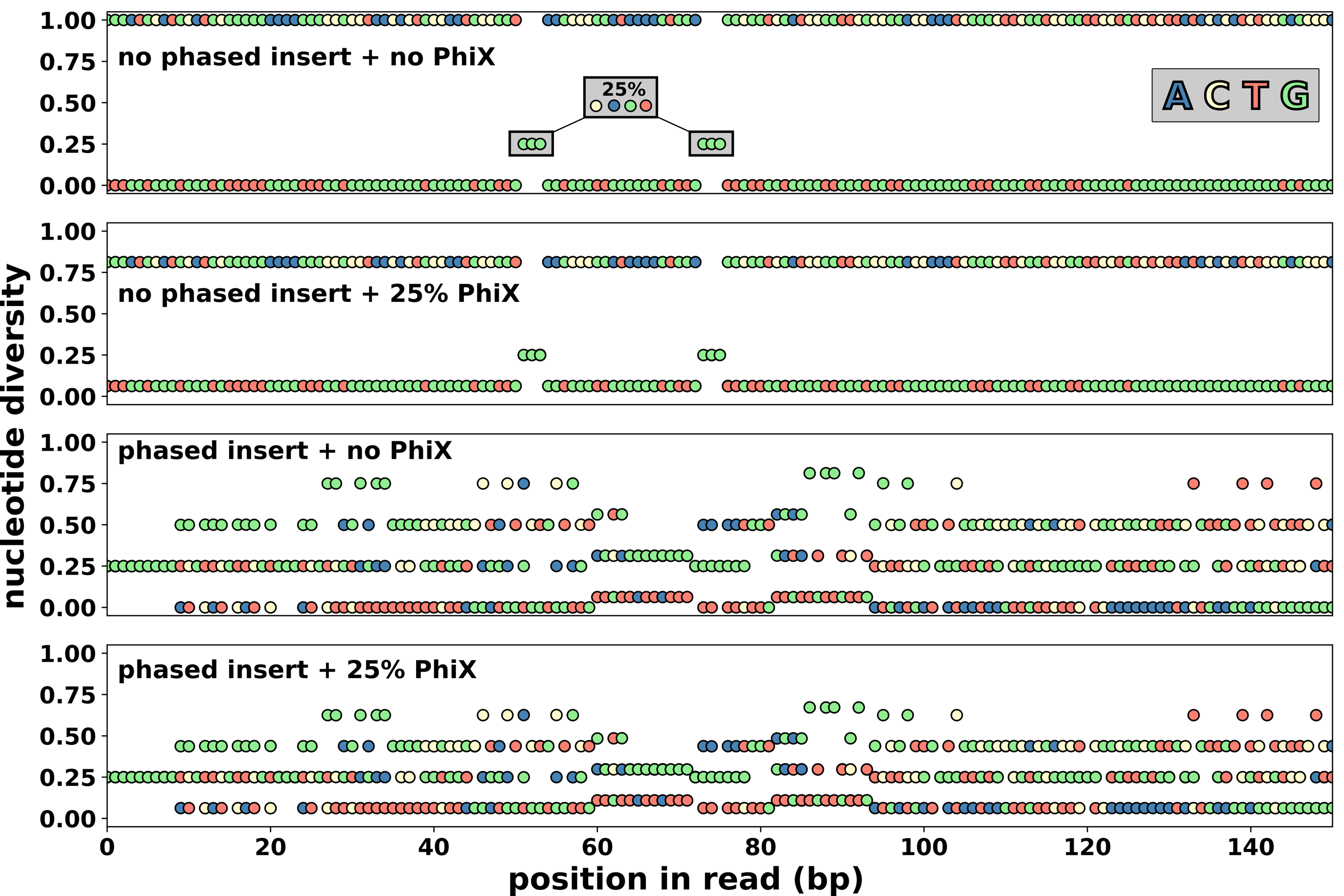
**

**SUPPLEMENTAL FIGURE S1. Prediction of nucleotide diversity from simulated sequence run.** Nucleotide diversity was predicted for four simulated twister ribozyme library samples. Predicted nucleotide diversity is shown for a control library without phased nucleotide insertions or PhiX, *only* 25% PhiX addition, only phased nucleotide insertions, or both phased insertions and PhiX. Each circle indicates the relative proportion of that nucleotide that is present at the position.


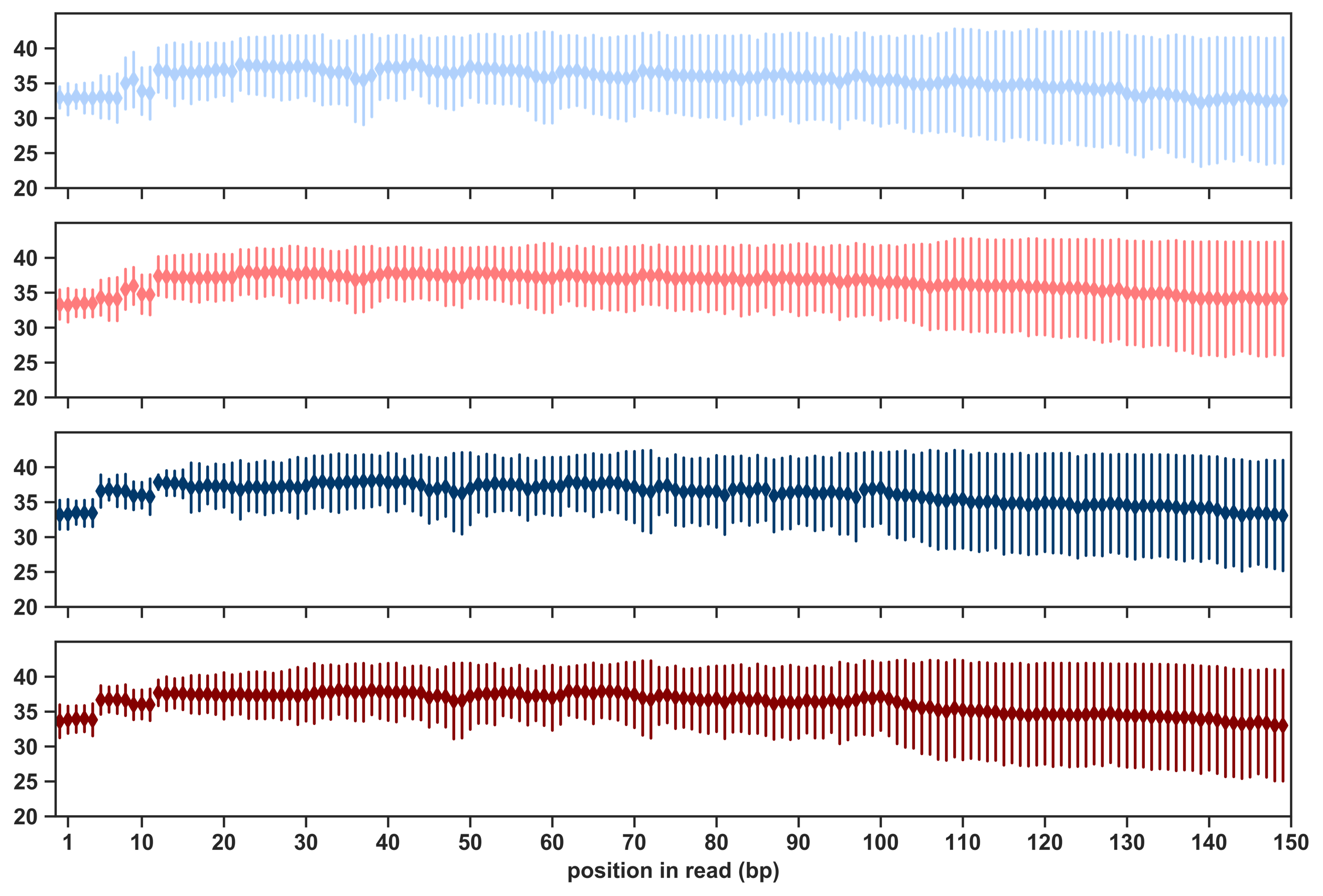


**SUPPLEMENTAL FIGURE S2.** Mean and standard deviation of sequencing quality scores per position in the read for samples without phased nucleotide inserts or PhiX (light blue), *only* 25% PhiX (light red), addition of only phased nucleotide insertions (dark blue), or both phased insertions and PhiX (dark red). Means are plotted and directly compared in Figure 3.

**
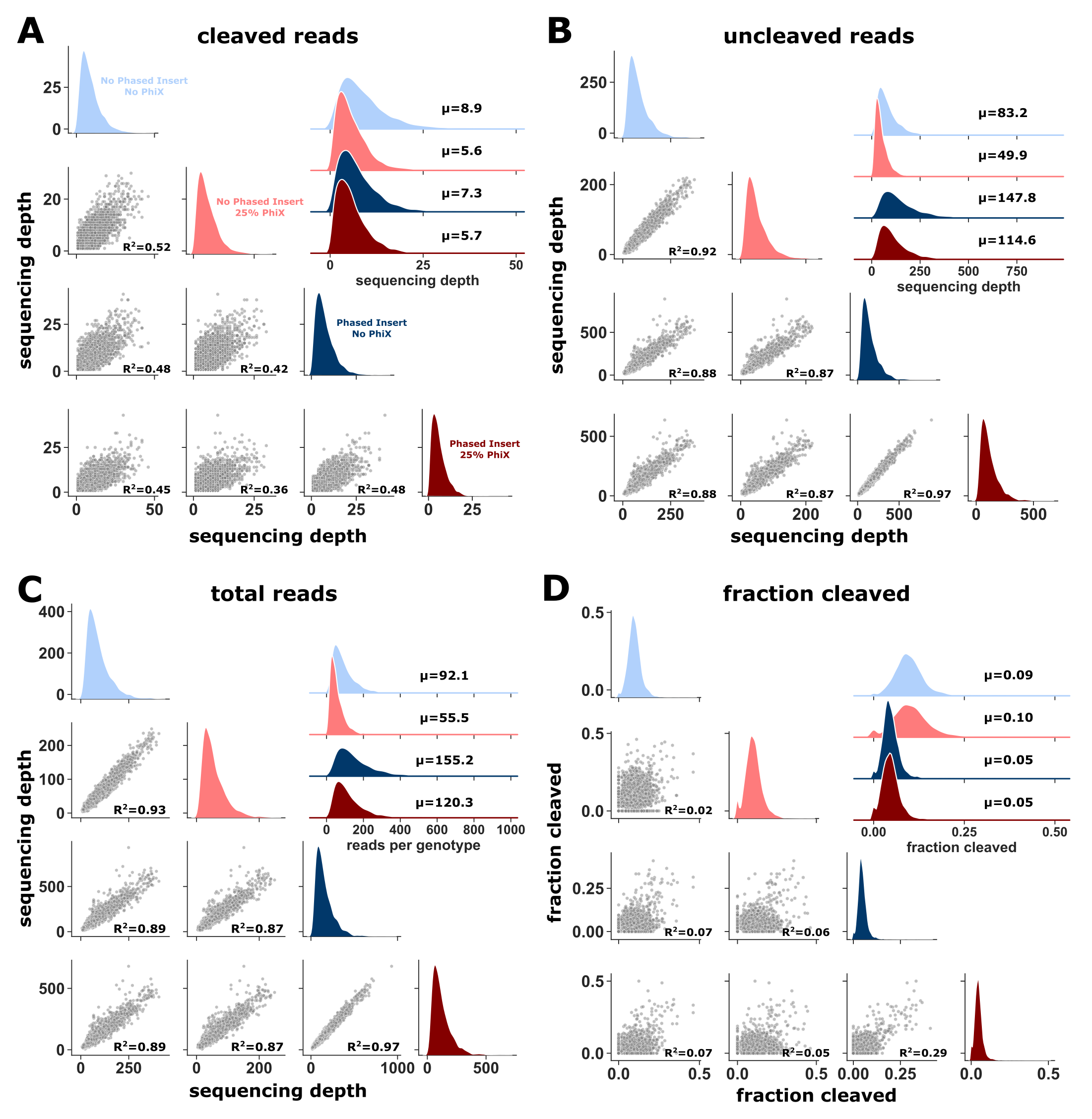
**

**SUPPLEMENTAL FIGURE S3.** **Comparison of sequencing depths and fraction cleaved between libraries.** ***(A)*** Correlation between cleaved reads per genotype observed in pairwise comparisons of the sequencing runs. Each unique sequence (n = 4,096) from the library is plotted as the number of sequencing reads found in that data set. Inset depicts the mean and distribution of cleaved reads. ***(B)*** Correlation between uncleaved reads per genotype observed in pairwise comparisons of the sequencing runs. Inset depicts the mean and distribution of uncleaved reads. ***(C)*** Correlation between total reads (cleaved + uncleaved) per genotype observed in pairwise comparisons of the sequencing runs. Inset depicts the mean and distribution of total reads. ***(D)*** Correlation between fraction cleaved per genotype observed in pairwise comparisons of the sequencing runs. Inset depicts the mean and distribution of fraction cleaved.

**
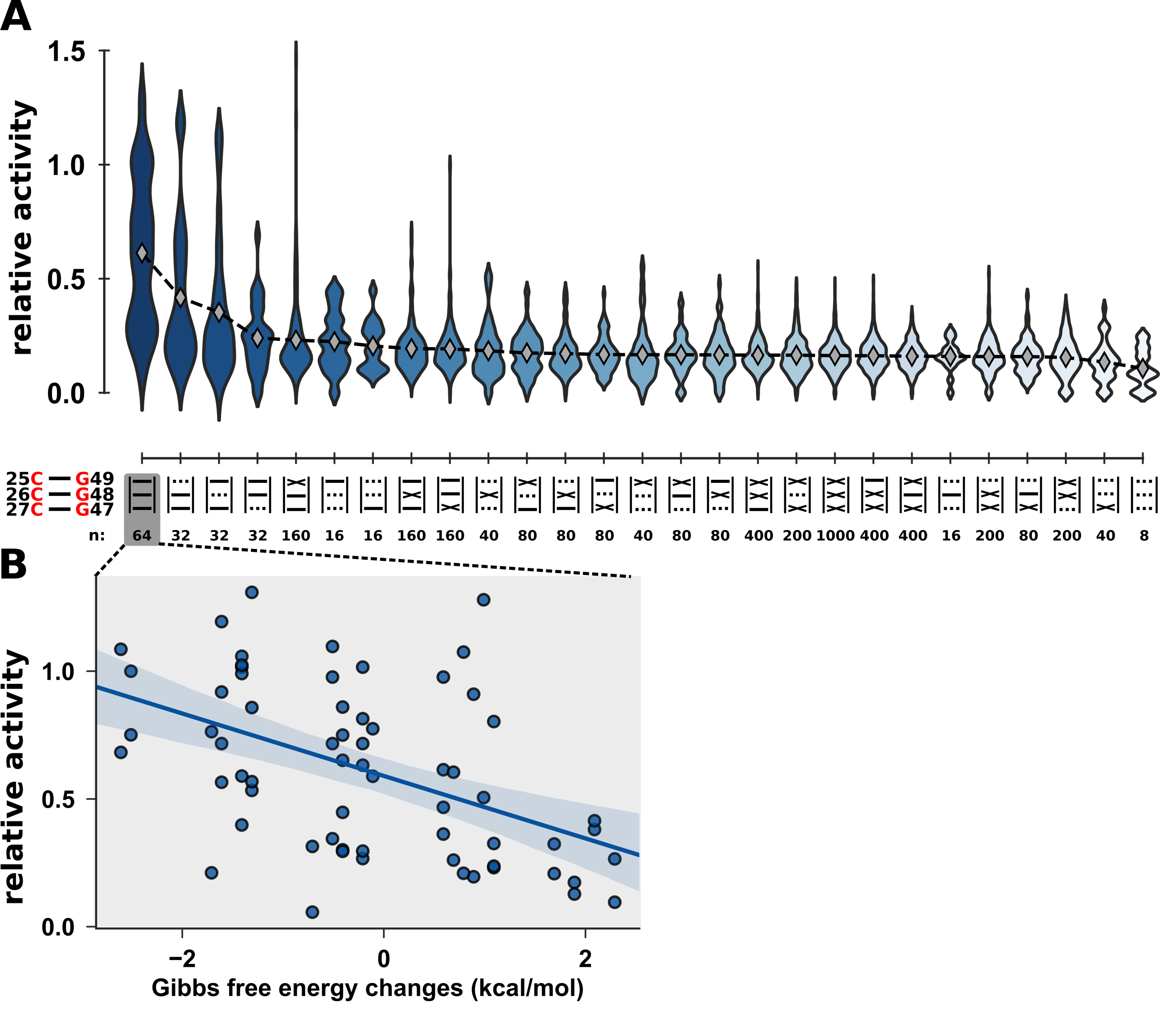
**

**SUPPLEMENTAL FIGURE S4. Relative activities from ribozymes categorized by the composition of base pairs in the T1 pseudoknot. *(A)*** Symbols on the categorical axis indicate Watson-Crick base pair (solid line), G-U wobble pairs (dashed lines) or mismatch (X). The number *n* below indicates the number of variants in each subpopulation. Dashed line and grey diamonds indicate the mean of each subpopulation. Data is rank ordered by the mean of the relative activity for the category. ***(B)*** Gibbs free energy changes for the 64 sequences that form three canonical base pairs. Gibbs free energy is calculated from the Nearest Neighbor Database (Turner and Mathews 2010). Line indicates the regression model with 95% confidence interval.
